# A discrete choice experiment to identify the most efficient quality indicators for the supervision of psychiatric hospitals

**DOI:** 10.1186/s12913-020-4993-1

**Published:** 2020-03-12

**Authors:** Pieter van Dijk, Ron Schellings, Brigitte A. B. Essers, Alfons G. Kessels, Ian Leistikow, Maurice P. Zeegers

**Affiliations:** 1 Dutch Health and Youth Care Inspectorate, Ministry of Health, Welfare, and Sport, Stadsplateau 1, 3521 AZ Utrecht, the Netherlands; 2 CAPHRI Care and Public Health Research Institute, Maastricht University, Maastricht, the Netherlands; 3 Department of Clinical Epidemiology and Medical Technology Assessment, Maastricht University Medical Center, Maastricht, the Netherlands; 4 Horten Centre, Zürich University, Zürich, Switzerland; 5 Institute of Health Policy and Management (iBMG), Erasmus University Rotterdam, Rotterdam, the Netherlands; 6 NUTRIM School of Nutrition and Translational Research in Metabolism, Maastricht University, Maastricht, the Netherlands

**Keywords:** Risk assessment, Discrete choice experiment, Health care regulatory agencies, Indicators, Mental health care, Quality and safety, Risk-based supervision

## Abstract

**Background:**

In the Netherlands, health care is regulated by the Health and Youth Care Inspectorate. Forty-six indicators are used to prioritize supervision of psychiatric hospitals. The objective of this study is to define a smaller set of weighted indicators which reflects a consensus among inspectors about which aspects are most important for risk assessment.

**Methods:**

The set of 46 indicators, complemented with missing information, was reduced to six indicators by means of interviews, group discussions and ranking among the inspectors. These indicators were used as attributes in a discrete choice experiment (DCE) to define their weights.

**Results:**

Twenty-six inspectors defined the top four indicators suitable for the risk assessment of psychiatric hospitals. These are: the policy on prevention of compulsory treatment; the policy on dysfunctional professionals; the quality of internal research after a serious incident; and the implementation of multidisciplinary guidelines on suicidal behaviour. These indicators share the same importance with regard to risk assessment. The screening of somatic symptoms and the policy on integrated care are important indicators too, but less relevant.

**Conclusion:**

Through a DCE, we reduced the amount of information for risk assessment of psychiatric hospitals to six weighted indicators. Inspectors can use these indicators to prioritize their inspections.

## What does this research introduce which is new?

- A limited set of weighted quality indicators can be applied for the risk assessment of psychiatric hospitals.
- This set is based on the preferences of the Health and Youth Care Inspectorate staff.
- A discrete choice experiment is an efficient method for reducing the amount of information required for supervision based on risk.
- Other stakeholders will be involved to define the most important indicators for risk assessment on mental health care.
- This, in turn, enhances the transparency and efficiency of supervision

## Background

It is the task of a government’s regulatory bodies, such as its inspectorates and market regulators, to ensure a satisfactory level of compliance with law and regulations, risk management and quality assurance. Although explicit definitions of regulation are scarce, a shared conception of what regulation entails has been identified. It is: the intentional intervention in the activities of a target population, where the intervention is typically direct. This involves setting binding standards, monitoring, and sanctioning, this later element being exercised by the public sector over the activities of the private sector [1]. In health care, the number of standards related to the quality of care has increased significantly over past decades. So too has the amount of information available to monitor health care providers [2]. For health care regulators, a major challenge is to identify those standards that have the most impact upon the quality of care and to identify the information that is most relevant for monitoring quality. This is important not only for the effective use of the limited resources of the regulator, but also for limiting the administrative burden regulation poses on health care organizations [3].

In the Netherlands, healthcare is regulated by the Health and Youth Care Inspectorate.

Since the turn of the century, this inspectorate has worked on developing new forms of supervision to face this challenge. One of these new approaches is risk-based supervision. This means that inspections are performed using the best indicators for predicting the quality and safety of health care. They intended to prioritize inspections by identifying health care institutions at greatest risk of failing in the quality of care they provide. However, until now, the choice of which institution to inspect is usually based on an assessment of a large set of pre-determined indicators. This is especially true for mental health care, which is one of the areas supervised by the Health and Youth Care Inspectorate. Here, indicators might not be considered to be measures of performance but rather to indicate a potential concern.

In the Netherlands, there are about 1300 specialized mental health care institutions including large integrated establishments with many locations and a large number of smaller ones [4]. Compulsory admission and treatment is permitted by law in 453 institutions and locations, including psychiatric hospitals, addiction clinics, wards of general and university hospitals, child/adolescent psychiatric institutions and forensic institutions [5]. The supervision of mental health care is performed by inspectors. Their decision to visit a psychiatric hospital for the assessment of the quality and safety of health care is based on a risk assessment using a set of 46 indicators [online Appendix A]. These indicators include information which it is mandatory for hospital boards to provide such as, process indicators on quality and safety, information on serious incidents, patients’ complaints and information from previously conducted inspections.

This amount of information is important in providing the context for inspectors.

However, it is not known what importance inspectors assign to these indicators and if there is any consensus about which indicators are deemed most important by the inspectors on which to base their choices [6].

The objective of this study is to define a limited set of weighted indicators, which reflects health care inspectors’ consensus on which aspects are most important to ensure the overall quality and safety of mental health care - and thus, which information is most suitable for risk assessment.

## Methods

### A discrete choice experiment

A discrete choice experiment (DCE) was conducted in order to rank indicators with respect to their importance and to enhance the transparency of the inspections being performed. A DCE is an attribute-based method for conducting surveys within health care in order to measure benefits, utility, or preference [7, 8].

It involves presenting respondents with a number of choice sets, which consist of two or more hypothetical alternatives that differ in their levels of various attributes. Every time a choice set is offered, respondents are asked to choose their preferred alternative. As such, a DCE forces respondents to make a trade-off between the levels of the different attributes when choosing an alternative. A crucial step in this experiment is the choice of the attributes that will determine, to a great extent, the degree to which the results can be applied elsewhere [9, 10].

### Establishing attributes

The selection of the attributes for the DCE was based on 46 indicators that were used by inspectors to prioritize their inspections in order to judge the quality and safety of mental health care. Firstly, seven inspectors were asked, one at a time, which of the 46 indicators could provide the best information for introducing priorities into the process of inspections and which indicators were lacking for this purpose. This resulted in a list of 17 indicators, of which nine were not among the original set of 46 indicators [see online Appendix B]. Secondly, these 17 indicators were discussed in five groups by 28 inspectors. Each group was asked which of the indicators could provide the best information to prioritize inspections and which indicators were lacking for this purpose. This resulted in a list of eight indicators, with four of them not in the original set of indicators. Thirdly, the same 28 inspectors ranked the three most important and the three least important indicators out of these eight indicators. They added no important additional indicators (see online Appendix C). The six indicators with the highest score were included as attributes for the DCE. Table 1 shows the attributes and the definitions.

**Table 1:** The attributes for the discrete choice experiment and their definitions

	Attribute	Definition
1	Policy regarding dysfunctioning professionals)^a^	The board conducts formalized policy to detect, promptly, dysfunctional employees and takes measures to stop the malfunction.
2	Quality of internal research after a serious incident	The board ensures that serious incidents are investigated in a systematic, self-critical and independent way and takes responsibility for implementing improvement measures.
3	Prevention of compulsory treatment	Policy to reduce compulsory treatment and convert compulsion into intensive care is defined and implemented.
4	Policy on integrated care^a^	The board has formalized partnerships with chain- and network partners in mental health care.
5	Screening of and treatment of somatic symptoms	Policy is implemented for screening patients for somatic symptoms frequently and for treatments of these problems.
6	Implementation multidisciplinary guidelines on suicidal behaviour^a^	The board facilitates the implementation of these guidelines and professionals comply with these guidelines if indicated.

^a^ not presented in the original set of 46 indicators

### Establishing levels

For each of these six attributes, two levels were defined: operational and not operational. The operational level was defined as fulfilling the conditions that all employees of mental health care institutes are well informed about the policy regarding an attribute of care, the policy is fully applied and the effects of the policy are periodically evaluated. Not operational was defined as there being no apparent policy.

### Experimental design

Using the rankings of the six indicators, an ‘efficient forced choice design’ of 32 choice sets was created by Ngene software version 1.1.2 (http://choice-metrics.com). The forced choice means that to opt-out was not an option. The 32 choice sets were presented in two versions of 16 by using blocking. An example of a choice set with the six attributes as used in the DCE is presented in Table 2.

**Table 2:** Example of a choice set with different profiles for two hypothetical psychiatric institutes

Attributes (variable label)	Psychiatric institute A	Psychiatric institute B
Screening for somatic symptoms	Operational	Not operational
Policy on integrated care	Operational	Not operational
Quality of internal research after a serious incident	Not operational	Operational
Prevention of compulsory treatment	Operational	Not operational
Implementation of multidisciplinary guidelines on suicidal behaviour	Not operational	Operational
Policy regarding dysfunctioning professionals	Not operational	Operational
Which hospital can assure best quality of care?	Ο A	Ο B

### Data collection

Twenty-nine employees of the Health and Youth Care Inspectorate, involved in the inspection of mental health care services, were asked to participate in the DCE. It concerned 19 inspectors with experience in patient care, eight co-inspectors with a background in health care, and two advising psychiatrists. They received the web-based questionnaire with general information and instructions. The questionnaire consisted of 16 choice sets with unlabeled descriptions of two fictional psychiatric hospitals A and B, which differed in their profiles in as much as they had different levels of the six attributes. Each time a choice set was offered, the respondent had to choose which hospital, A or B, provided the best quality of care. We used an unlabeled generic design, which means that the labels attached to each option convey no information beyond that provided by the attributes [8].

As contextual information, it was stated that the delivery of care and the organization of the hospital were the same in all remaining aspects. The order of the attributes within the scenarios was assigned randomly to the three groups of respondents in such way that no group of respondents was offered the same order.

### Statistical analysis

The weights of the attributes were determined with a logistic regression model for panel data with the choice of the respondents as the dependent variable and the attributes as independent variables. Effect coding was used with the levels of the attributes set to − 1 or 1 with the constant set to zero. The exponent of the regression coefficient of a specific attribute in the model (odds ratio) can be interpreted as the relative influence on the decision compared to the influence of the reference attribute that was left out. The significance levels of the differences of all possible pairs of coefficients were determined.

## Results

### Response

Twenty-six employees (93%) participated in the DCE, consisting of 18 inspectors, six co-inspectors, and two psychiatrist inspectors. All had worked at the Inspectorate from one to 34 years with a median of 8 years (25% percentile =2.75; 75% percentile = 14.25). The three employees, who did not respond, did not significantly differ with regard to the duration of work experience compared to the respondents.

### Weight of attributes

Table 3 gives an overview of the relative influences (odds ratios) of the different attributes.

**Table 3:** Odds Ratios as a measure of the relative importance of the six attributes selected and the significance of the differences of these odds ratios (pairwise comparison)

Attributes (variable label)	Odds Ratio	95% CI	Pairwise comparison of the odds ratios				
			Dysfunc	Intern	Guide	Screening	Network
Prevention compulsory treatment (Compuls)	3,96	2.88–5.44	n.s.	n.s.	n.s.	¶	¶
Policy dysfunctional professionals (Dysfunc)	3,94	2.85–5.45		n.s.	n.s.	¶	¶
Quality internal research (Intern)	3,85	2.70–5.49			n.s.	¶	¶
Implementation guidelines suicidal behaviour (Guide)	2,99	2.07–4.32				§	§
Screening/treatment somatic symptoms (Screening)	2,04	1.46–2.84					n.s.
Policy integrated care (Network)	1,95	1.46–2.84					

n.s.: the difference between odd ratios is not statistically significant

§: the difference between odd ratios is statistically significant, *p* < 0.01

¶: the difference between odd ratios is statistically significant, *p* < 0.05

An odds ratio equal to 1 means that the attribute in question is not relevant for determining whether or not a psychiatric hospital can assure the best quality of care. For all six attributes the minimum of the 95% confidence interval around the odds ratio is higher than 1. That means that according to the respondents, all attributes are indeed important. Attributes with higher odds are more relevant. According to the respondents a psychiatric hospital with an operational policy aimed at preventing compulsory treatment has a four times higher probability to assure best quality of care than a psychiatric hospital without such operational policies. The same is true for the attributes policy on dysfunctional professionals and the quality of internal research after a serious incident. The screening for somatic symptoms and the policy on integrated care seem to be less important indicators for assessing the quality and safety of care in a psychiatric hospital.

The four highest odds ratios do not differ significantly from each other. However, they are significantly higher than the odds ratio of the last two attributes.

In general, the six attributes reflect the top six indicators derived from the 46 indicators already in use by inspectors and the nine added indicators, which are deemed to be important for the assessment of the quality and safety of mental health care.

## Discussion

The primary objective of this study was to reduce the set of 46 indicators used for the risk assessment of the quality and safety of mental health care, provided by psychiatric hospitals, to a limited set of weighted indicators. We reduced successfully, a large set of unweighted indicators into a small set of six weighted indicators. This set is based on a consensus among the inspectors.

From the perspective of the Health and Youth Care Inspectorate, there are four top indicators suitable for supervision on Dutch mental health care based on risk. These are the policy for the prevention of compulsory treatment, the policy on dysfunctional professionals, the quality of internal research after a serious incident and the implementation of multidisciplinary guidelines on suicidal behaviour. These indicators share the same importance for the assessment of quality and safety of mental health care. The screening for somatic symptoms and the policy on integrated care are important indicators too, but less relevant.

The degree to which these indicators can be applied depends on the data which boards of directors are mandated to provide. They are responsible for the quality and safety of care provided in psychiatric hospitals. Thereafter, inspectors can use the information on these indicators to determine whether a psychiatric hospital should or should not be visited. Obviously, additional information is available for this decision, but these indicators appear to be the most important for this risk based assessment. Visits can lead to in-depth supervision, checks on the validation of the data provided and discussions on the data with the board of directors.

In practice of the supervision by the Health and Youth Care Inspectorate the indicators could be assessed by means of a web-based survey (the degree of implementation multidisciplinary guidelines on suicidal behaviour and the degree of screening of and treatment of somatic symptoms), by means of mandatory information provided by hospital boards (data about compulsory treatment, reports about internal research after a serious incident and notifications of malfunctioning professionals) and by means of conducted inspections (policy on integrated care).

A limitation of this study is that the results only concern the consensus and opinions of the inspectors about to the importance of the indicators. They do not involve the boards of directors, responsible for the quality and safety of care provided in psychiatric hospitals and who are under supervision. Therefore, more parties should be involved in the development of quality indicators for the regulation of health care. These could include boards of directors, supervisory boards and the patient’s councils of psychiatric hospitals. This will enhance the transparency and efficiency of risk-based supervision and might lead to shared interests about the use of quality indicators.

Another limitation is that in this discrete choice experiment attributes are formulated dichotomously. Attributes are “on or off”; policy is fully present of completely absent. In ‘real life’, there are gradations and grey areas. Risk assessment must take this into account.

Our study was restricted to the regulation of Dutch mental health care. However, this method could also be applied to a great many other fields of health care from the point of view of the regulations. In addition, an international approach to the development of quality indicators for the regulation of health care is also important [9–11].

We used a DCE for selecting quality indicators in this study. In general, the external validity of the results of a DCE is considered limited since the group of respondents is often a small sample of the target population. However, in this study the selection of respondents consisted of all the inspectors who are involved in the regulation of hospitals providing mental health care. This means that in total 29 persons were invited to participate in the DCE. Although three persons did not respond, their characteristics did not differ from those respondents who participated in the DCE.

To the best of our knowledge, this is the first time that a large set of unweighted indicators were refined to a small set of weighted indicators for the regulation of care provided by psychiatric hospitals by means of a DCE. In 2012 we used a DCE to examine which health care indicators are considered the most important in the assessment of the quality of care for patients with schizophrenia and drug dependency [6]. The results of a study using a DCE might contribute to the development of a uniform and consistent method for regulatory agencies to focus their inspections on high risk health services. In addition, this enables them to provide feedback to health services and hospitals and to offer a meaningful contribution to improvement in the quality of care and to patient’s safety.

## Conclusions

Using a discrete choice experiment, we reduced a large amount of information for risk assessment of psychiatric hospitals to a small set of six weighted indicators concerning:

- the policy for the prevention of compulsory treatment;
- the policy on dysfunctional professionals;
- the quality of internal research after a serious incident;
- the implementation of multidisciplinary guidelines on suicidal behaviour;
- the screening for somatic symptoms and the policy on integrated care.

Inspectors can use these indicators to prioritize their inspections. These indicators are based on their preferences. Preferences of other stakeholders, such as Boards of Directors and (representatives of) clients in mental health care should be investigated to determine whether these are indeed the most important indicators for risk assessment in mental health care. This, in turn, enhances the transparency and efficiency of supervision.

## Supplementary information

**Additional file 1. **Online **Appendix A**: 46 pre-determinated indicators for risk-based supervision of Dutch psychiatric hospitals. Online **Appendix B**. List of indicators defined in the process of establishing attributes for the discrete choice experiment. Online **Appendix C**. Short list of indicators for risk based supervision of Dutch psychiatric hospitals, defined in the process of establishing attributes for the discrete choice experiment.

## Data Availability

The datasets generated and/or analyzed during the current study are available from the corresponding author on reasonable request.

## Abbreviations

CAHPRI: Care and Public Health Research Institute, Maastricht University
DCE: Discrete Choice Experiment
iBMG: Institute of Health Policy and Management, Erasmus University Rotterdam
NUTRIM: School of Nutrition and Translational Research in Metabolism, Maastricht University
