# Supplementary material for: A discrete choice experiment to identify the most efficient quality indicators for the supervision of psychiatric hospitals
Source: BMC Health Serv Res. 2020 Mar 12;20:192. doi: 10.1186/s12913-020-4993-1 (PMC7069034; doi:10.1186/s12913-020-4993-1)
Supplement: Supplementary file 1 — Additional file 1. Online Appendix A: 46 pre-determinated indicators for risk-based supervision of Dutch psychiatric hospitals. Online Appendix B. List of indicators defined in the process of establishing attributes for the discrete choice experiment. Online Appendix C. Short list of indicators for risk based supervision of Dutch psychiatric hospitals, defined in the process of establishing attributes for the discrete choice experiment. [file 12913_2020_4993_MOESM1_ESM.docx]

**ONLINE APPENDIX A:**

**46 pre-determinated indicators for risk-based supervision of Dutch psychiatric hospitals.**

|  | **Short description** | **Referred aspect** | **Data source** | **attribute for DCE** |
| --- | --- | --- | --- | --- |
| 1 | General judgement on quality and safety | Inspectors judgment | Supervision | No |
| 2 | Quality of internal research after a serious incident | Inspectors judgment | Supervision | Yes |
| 3 | Compliance governance code | Governance | Corporate information | No |
| 4 | Absenteeism | Governance | Corporate information | No |
| 5 | Leaving staff | Governance | Corporate information | No |
| 6 | Entering staff | Governance | Corporate information | No |
| 7 | Submitted complaints | Governance | Corporate information | No |
| 8 | Liquidity | Finance | Corporate information | No |
| 9 | Capital buffer | Finance | Corporate information | No |
| 10 | Degree of solvency | Finance | Corporate information | No |
| 11 | Debt coverage ratio | Finance | Corporate information | No |
| 12 | Profit ratio | Finance | Corporate information | No |
| 13 | Total number of beds | Context | Corporate information | No |
| 14 | Number of beds psychiatric ward | Context | Corporate information | No |
| 15 | Number of clients | Context | Corporate information | No |
| 16 | Total full time equivalent (FTE) of staff | Context | Corporate information | No |
| 17 | FTE staff psychiatric ward | Context | Corporate information | No |
| 18 | Quality of medical care in detention centers | Care-related | Performed supervision on actual themes | No |
| 19 | Quality of public mental health care | Care-related | Performed supervision on actual themes | No |
| 20 | Somatic co-morbidity | Care-related | Performed supervision on actual themes | Yes, part of attribute “screening on somatic symptoms” |
| 21 | Reducing separations | Care-related | Performed supervision on actual themes | Yes, part of attribute "prevention of compulsory treatment” |
| 22 | Heroin-methadone | Care-related | Performed supervision on actual themes | No |
| 23 | Medication safety | Care-related | Performed supervision on actual themes | No |
| 24 | Outpatient geriatric care | Care-related | Performed supervision on actual themes | No |
| 25 | Number of suicides | Care-related | Calamities reports | No |
| 26 | Number of suicide attempts | Care-related | Calamities reports | No |
| 27 | Measuring severity of psychiatric problems | Care-related | Basic set of risk indicators | No |
| 28 | Screening on somatic problems | Care-related | Basic set of risk indicators | Yes, part of attribute “screening for somatic symptoms” |
| 29 | Screening for somatic problems long-stay clients | Care-related | Basic set of risk indicators | Yes, part of attribute “screening for somatic symptoms” |
| 30 | Contact after discharge | Care-related | Basic set of risk indicators | No |
| 31 | Clinical report of medications | Care-related | Basic set of risk indicators | No |
| 32 | Outpatient report of medications | Care-related | Basic set of risk indicators | No |
| 33 | Number of separations per 1,000 inpatient days | Care-related | Basic set of risk indicators | Yes, part of attribute "Prevention of compulsory treatment” |
| 34 | Length of separations | Care-related | Basic set of risk indicators | Yes, part of attribute "Prevention of compulsory treatment” |
| 35 | Number of separations per 1,000 inpatient days | Forensic psychiatry | Basic set of risk indicators | Yes, part of attribute "Prevention of compulsory treatment” |
| 36 | Length of separations | Forensic psychiatry | Basic set of risk indicators | Yes, part of attribute "Prevention of compulsory treatment” |
| 37 | Number of separations per client | Forensic psychiatry | Basic set of risk indicators | Yes, part of attribute "Prevention of compulsory treatment” |
| 38 | Number of separations per separated client | Forensic psychiatry | Basic set of risk indicators | Yes, part of attribute "Prevention of compulsory treatment” |
| 39 | Length of separations | Forensic psychiatry | Basic set of risk indicators | Yes, part of attribute "Prevention of compulsory treatment” |
| 40 | Somatic screening admission | Forensic psychiatry | Basic set of risk indicators | Yes, part of attribute “screening on somatic symptoms” |
| 41 | Somatic screening long-term admissions | Forensic psychiatry | Basic set of risk indicators | Yes, part of attribute “screening on somatic symptoms” |
| 42 | Number of complaints | Care-related:  Counselor | Counselor report | No |
| 43 | Number of asked question | Care-related:  Counselor | Counselor report | No |
| 43 | Formal procedures | Care-related:  Counselor | Counselor report | No |
| 44 | Signals to Board | Care-related:  Counselor | Counselor report | No |
| 45 | Reactions of Board | Care-related:  Counselor | Counselor report | No |
| 46 | Top three complaints | Care-related:  Counselor | Counselor report | No |

**ONLINE APPENDIX B**

**List of indicators defined in the process of establishing attributes for the discrete choice experiment**

|  | **Short description** | **Referred aspect** | **Data source** | **Attribute for DCE** |
| --- | --- | --- | --- | --- |
| 1 | Quality internal research after a serious incident | Inspectors judgment | Supervision | Yes |
| 2 | Submitted complaints *(and reaction of the board to substantiated complaints)* | Governance | Corporate information | No |
| 3 | Reports of counselor *(and reaction of the board to complaints)* | Care-related:  Counselor | Counselor report | No |
| 4 | Presence of a family council | Context | New indicator to be developed | No |
| 5 | Policy on dysfunctional professionals | Governance | New indicator to be developed | Yes |
| 6 | Policy regarding ongoing training of staff | Governance | New indicator to be developed | No |
| 7 | Policy on integrated care | Governance | New indicator to be developed | Yes |
| 8 | The outcomes of monitoring themes | Care-related themes | Supervision | No |
| 9 | Number and severity of reported fire-, shooting-, stabbing- and other incidents of aggression | Context | New indicator to be developed | No |
| 10 | Number and severity of reported dysfunctional professionals | Governance | New indicator to be developed | Yes, part of attribute “policy on dysfunctional professionals”. |
| 11 | Number and severity of reported incidents of somatic comorbidity | Care-related | New indicator to be developed | Yes, part of attribute “screening for somatic symptoms” |
| 12 | Number of suicides (attempts) | Care-related | Calamities reports | No |
| 13 | Prevention of compulsory treatment and deployment of intensive care | Care-related | Basic set of risk indicators | Yes |
| 14 | Outpatient medication safety | Care-related | Basic set of risk indicators | No |
| 15 | Implemented multidisciplinary guideline for diagnosis and treatment of suicidal behaviour | Care-related | New indicator to be developed | Yes |
| 16 | Policy to reduce aggression risks | Governance | New indicator to be developed | No |
| 17 | Screening for somatic symptoms | Care-related | Basic set of risk indicators | Yes |

**ONLINE APPENDIX C**

**Short list of indicators for risk based supervision of Dutch psychiatric hospitals, defined in the process of establishing attributes for the discrete choice experiment**

|  | **Short description** | **Referred aspect** | **Data source** | **Attribute for DCE** |
| --- | --- | --- | --- | --- |
| 1 | Quality internal research after a serious incident | Inspectors judgment | Supervision | Yes |
| 2 | Submitted complaints *(and reaction of the board to substantiated complaints)* | Governance | Corporate information | No |
| 3 | Policy on dysfunctional professionals | Governance | New indicator to be developed | Yes |
| 4 | Policy on integrated care | Governance | New indicator to be developed | Yes |
| 5 | Prevention of compulsory treatment and deployment of intensive care | Care-related | Basic set of risk indicators | Yes |
| 6 | Outpatient medication safety | Care-related | Basic set of risk indicators | No |
| 7 | Implemented Multidisciplinary Guideline for diagnosis and treatment of suicidal behaviour | Care-related | New indicator to be developed | Yes |
| 8 | Screening for somatic symptoms | Care-related | Basic set of risk indicators | Yes |
